# Utilization of optimized microwave sintering to produce safe and sustainable one-part alkali-activated materials

**DOI:** 10.1038/s41598-023-31581-0

**Published:** 2023-03-21

**Authors:** Moataz Refaat, Alaa Mohsen, El-Sayed A. R. Nasr, Mohamed Kohail

**Affiliations:** Faculty of Engineering, Ain Shams University, Cairo, 11517 Egypt

**Keywords:** Chemical engineering, Civil engineering

## Abstract

Sodium hydroxide (NaOH) as an alkaline activator presents a vital limitation in the mass production of alkali-activated binders due to its severe effect on users’ safety. In this study, safe and sustainable one-part alkali-activated slag mixes (OP-AAS) were prepared through an efficient microwave sintering for a mixture of active amorphous ground granulated blast furnace slag (GGBFS) and sodium hydroxide powder (NaOH). Different microwave-sintered powders were prepared using microwave energy of power 900 W for the mixture at different treatment periods (10, 20, and 30 min). Fresh and hardened properties of different OP-AAS mixes were studied. Moreover, the phase composition and microstructure were investigated using X-ray diffraction (XRD) analysis and scanning electron microscope (SEM). Cytotoxicity/viability testing was performed to evaluate the cell death induced by the developed materials to measure their safety for the user. According to compressive strength, cytotoxicity/viability analysis, environmental impact and cost calculation of developed OP-AAS, it is concluded that employing microwave sintering for a short duration is sufficient to produce safe binding materials with adequate mechanical properties suitable for commercial applications in the construction sector.

## Introduction

**Figure 1:**
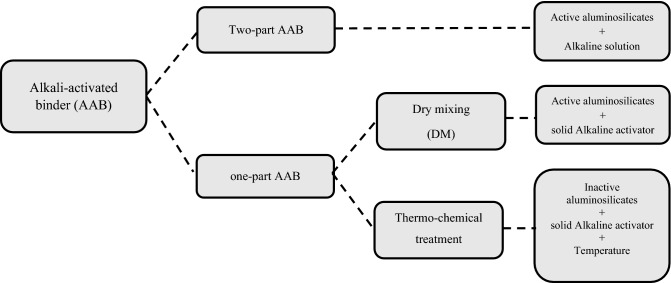
Alkali-activated binder production classification.

Alkali-activated binders (AABs) have been studied widely as alternative binders to Portland cement (PC) in the concrete industry due to sustainability, environmental and economic considerations^1–5^. AABs are a sustainable alternative to PC, prepared from waste or by-product materials that help conserve natural resources utilized during the PC industry^6,7^. In addition, waste utilization saves the large area needed in case of disposal or storage^8–10^. Environmentally, AABs show a low environmental footprint compared to PC, which produces around (0.5 to 0.82) kg CO_2_ for every kg PC produced^11–15^. Economically, they are produced from low-cost materials (by-products) with no need for high energy consumption, in most cases, during manufacturing^16–18^. AABs are formed through a geopolymerization process resulting from mixing base materials containing a high percentage of silica and alumina (aluminosilicate source) in an alkaline medium (alkaline activator)^19–21^. The aluminosilicate source can be obtained from geological sources such as metakaolin (MK) or industrial by-products such as GGBFS from the steel production industry and fly ash (FA) from bituminous or anthracite coal combustion^22–24^. The commonly used alkaline activator can be sodium/potassium hydroxide (Na/KOH), silicate (Na_2_/K_2_Si_2_O_3_), carbonate (Na_2_/K_2_CO_3_), and oxide (Na_2_/K_2_O)^25,26^. After many studies on alkali-activated materials, AABs can be classified according to the production method into two main systems: a two-part AAB system and a one-part AAB system, as clarified in Fig. 1.

The traditional two-part AAB system is the principal technique for producing AABs in which the active amorphous aluminosilicate material is combined with the previously prepared strong alkaline solution^27–30^. Two-part AABs have significant properties; they show high mechanical properties in terms of compressive strength^31–33^, bond strength^30,34^, and withstanding fatigue loads^35,36^. Also, they have higher durability than PC in terms of resistance to acids^37–39^, chemicals^40,41^, freeze–thaw cycles^42,43^, and elevated temperature^44–46^. Despite the benefits of two-part AABs, the presence of the alkaline activator in a solution form is one of the main challenges that face their scalability due to transportation, mixing and placing difficulties of the concrete. Furthermore, some of the utilized alkaline solutions have economic and environmental problems.

One-part AAB system is a forward step in the large-scale production of AAB concrete as it is more useful and easier for in-situ applications than the two-part method, with a physical form that resembles the PC commercial form (one-part product)^25,26,47^. In addition, a one-part AAB system has low environmental footprint than a two-part AAB system. Luukkonen et al.^47^ reported that the environmental impacts for different one-part and two-part AABs were only 24% and 60% of the environmental impact of PC, which clarified the low environmental impact of the one-part AAB system compared to the two-part AAB system.

In one-part AABs, there are two approaches for producing AAB powder depending on the activity of the aluminosilicate base material. The first approach is the dry mixing (DM) of the solid alkaline activator with active amorphous aluminosilicates^48–50^; then, the reaction begins by just adding water. The solid alkaline activators used in this approach are mainly Na_2_Si_2_O_3_, NaOH, and KOH or a mixture of other solid activators. Preparing DM using Na_2_Si_2_O_3_ as a solid activator presents high mechanical properties compared to two-part AAB and lower alkalinity problems^26,51^. However, it faces economic and environmental challenges due to high activator costs and high carbon dioxide gases produced during activator manufacturing^52^. Yousefi Oderji et al.^53^ produced different DM mixes using a mixture of NaOH and KOH as a solid alkaline activator. However, the mixes suffered from difficult handling, poor flowability, and comparatively low mechanical properties due to the high amount of heat that evolved from the exothermic reaction. As reported in previous studies, the high heat evolved could be responsible for internal thermal strain; micro-cracks were formed^54,55^. In addition, NaOH and KOH alkaline activators are very dangerous to be used by users during the handling and mixing stages. Askarian et al.^56^ used different solid alkaline activators (Ca(OH)_2_, Na_2_O, Li(OH)_2_, K_2_CO_3_) combined with Na_2_Si_2_O_3_ to activate a mixture of slag and fly ash. Nevertheless, the results showed that a high activator percentage (27%) was used to achieve a compressive strength of 38 MPa, which was not economically and environmentally useful.

The second approach for producing one-part AAB is the thermo-chemical treatment process (TCT) of inactive aluminosilicate material through sintering (treatment) in the presence of a solid alkaline activator (e.g., NaOH, Na_2_CO_3_). This approach aims to increase the amorphousity (activity) of the aluminosilicate material to produce one-part AAB with good physical and mechanical properties^57–60^. Abdel-Gawwad et al.^58^ used thermo-chemical activation to make one-part AAB from cement kiln dust (CKD) and feldspar (FS) blends with a CKD/FS weight ratio of 60/40. In the presence of Na_2_CO_3_, the mixes were subjected to different thermal temperatures of 1200 °C and 1300 °C for 2 and 3 h. The compressive strength of a mix subjected to 1300 °C for 3 h in the presence of 20% Na_2_CO_3,_ by weight, as a solid alkaline activator, is 52 MPa. Although achieving high compressive strength values for the blends, the high energy consumed (1300 °C) and the high alkaline activator content represented economic and environmental problems. Liu et al.^61^ studied the effect of applying different temperature degrees (300, 500, 700 °C) on a mixture of inactive lithium slag (LS) and solid NaOH. The active amorphous constituents increased significantly from 17.3 to 50.7 wt.%, achieving a compressive strength of 50 MPa after heat activation of 700 °C. Abdel-Gawwad et al.^57^ applied a combination of NaOH alkaline activator and elevated thermal sintering of 1100 °C and 1200 °C to treat concrete waste and reuse it as a ready mix alkali-activated cement. This approach's main challenge is the high energy consumed during sintering, which is not environmentally and economically accepted. Although the thermo-chemical treatment process utilized an enormous amount of energy, no handling, flowability, or exothermic-microcrack problems were recorded in the case of the NaOH alkaline activator. This result could highlight a positive impact of the thermos-chemical treatment process in solving some of NaOH’s one-part AAB drawbacks. Abdel-Gawwad et al.^62^ used chemical treatment without applying high temperature by mixing concrete waste with NaOH, then mixing water and drying in the oven at 60 °C for 18 h. The treated mixture was ground and dry mixed with GGBFS. The compressive strength result of the hardened cubes after 120 days of curing was 29 MPa, which is a relatively low value.

Microwave heating is a recent technology used in cement and concrete industries with high interest and continuous improvement due to its many advantages^63–66^. Compared to conventional heating, microwave heating has a short operation cycle due to rapid heating rates with the short heating time required, safe, controlled operation due to instantaneous electronic control, and energy optimization through its’ volumetric and selective heating mechanism, which directly penetrates the material depending on its dielectric properties. Furthermore, it provides a clean heating process due to the absence of secondary waste generation^67–69^. The microwave heating process depends on absorbing the electromagnetic energy by the molecular bonds inside the material, then transforming it into heating energy by vibrating and exciting action. It was reported that microwave heating impacts materials with dielectric properties, such as cement and concrete, more than conventional heating^70^. Consequently, many researchers studied the use of microwave heating in different cementitious applications such as cement clinker production with low energy instead of heating using a rotary kiln at 1450 °C^71,72^; similar to autoclave it can be used in the fabrication of pre-cast concrete as it can accelerate the concrete curing process^73^, improving the interlayer bonding and buildability of geopolymer 3D concrete^74,75^, and others^76,77^. Although using microwave sintering in PC clinker production has many operating advantages, as previously mentioned, it does not offer any advantage on the scale of energy consumption^78^. Buttress et al.^79^ reported that the energy needed for PC clinker production in microwave heating is about (250–470%) of the energy used by the conventional method. This high energy consumption is due to the presence of calcium carbonate with a high percentage (80%) in PC clinker chemical composition, which is a poor microwave-heating absorber compared to iron (Fe_3_O_4_), aluminum (Al_2_O_3_) and silicon (SiO_2_) oxides. However, the presence of a high percentage of Al_2_O_3_ and SiO_2_ oxides in AABs qualifies the microwave heating of aluminosilicate materials to be more effective with low energy consumption^64,80^. Kim et al.^81^ reported that the existence of Al_2_O_3_ and SiO_2_ oxides with high content in clay-based materials guarantees high absorption of microwave energy and good heating and curing process. Accordingly, utilizing microwave heating in the AABs industry could be a promising research interest.

The environmental and economic limitations of two-part and one-part AAB systems are the main challenges that face their large-scale production. Hence, it was essential to find a reliable system that benefits from the advantages of both systems and avoids their drawbacks. To achieve such a system, it was essential to answer three main questions: (i) What the suitable commercial form of the product is; (ii) How to obtain relevant engineering properties and achieve user safety and (iii) How to create the environmental and economic balance. According to the literature illustrated above, manufacturing NaOH one part AAB by thermo-chemical treatment (TCT) could be a commercial product if its drawbacks are eliminated. Production of TCT mainly depends on three main factors: base material, alkaline activator and sintering/treatment condition. The base materials used in several studies by this technique were mainly inactive and crystalline materials such as feldspar, concrete waste, air-cooled slag and lithium slag, which needed an elevated temperature reached 1300 °C for a long curing period of 3 h, where the main purpose was to transform the crystalline (inactive) aluminosilicate precursor to amorphous (active) one. While in this study, amorphous materials such as GGBFS were used, where the main purpose of the sintering process is only embedding the NaOH into GGBFS and mitigating its severe effect on users’ safety. This advantage aided in reducing the sintering energy utilized, creating an environment-economic balance for the prepared product. Furthermore, all previous studies depended on using a conventional heating system in the thermal treatment process, which has a detrimental effect on the environment and human health. Consequently, a clean and efficient energy source, which is microwave sintering, was utilized for the sintering process. This work aims to benefit from microwave heating technology (low energy consumption and short treatment periods) in the presence of active aluminosilicate to produce safe and sustainable one-part alkali-activated materials with adequate mechanical properties. Microwave sintering for different periods was applied to the GGBFS/NaOH dry mixture and their effect on the mixes' fresh properties, hardened properties and the skin toxicity degree of the alkaline activator was investigated.

## Experimental program

### Materials selection criteria and characterizations

The materials used in this study are: (i) active amorphous obtained from steel production factories used as raw material, an industrial by-product (waste) with an amorphous microstructure supplied from Lafarge Company, Suez, Egypt. The chemical composition (using an X-ray fluorescence analyzer (XRF, Xios PW 1400)) has been tabulated in Table 1. While, Table 2 shows the physical properties of the used GGBFS. According to ASTM C989, the used GGBFS has an activity index of grade 100, referring to its moderate activity. (ii) Traditional NaOH pellets with a purity of 99% produced by Al-Ahram company, Giza, Egypt, was used as an alkaline activator.

**Table 1:** Oxides composition of GGBFS.

Oxides composition of GGBFS (wt%)									
SiO_2_	Al_2_O_3_	Fe_2_O_3_	CaO	MgO	MnO	TiO_2_	SO_3_	LOI	Total
35.40	17.40	1.40	36.87	6.83	0.35	0.11	0.24	0.5	99.1

**Table 2:** Physical Properties of GGBFS.

Physical properties of GGBFS	
Specific gravity	2.80
Bulk density (t/m^3^)	1.15
Specific surface area (cm^2^/gm)	4088

### Mix proportions, casting, and curing

**Figure 2:**
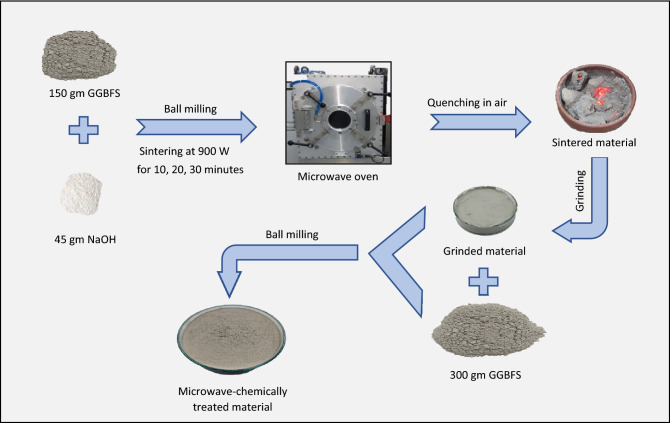
Preparation steps od microwave-chemically treated OP-AAS powder.

Five mixes were designed in this approach, as presented in Table 3. The total weight of the used GGBFS was 450 gm, and the weight of the NaOH pellets was 45 gm (10% of the total slag weight). Two-part alkali-activated slag (TP) is a traditional mix designed as a control mix to be compared with the newly developed mixes. TP was prepared by adding NaOH solution (pre-dissolving NaOH in the mixing water with a molarity of 9.2 M) to GGBFS powder. The second mix was a one-part dry mix (DM) prepared by mixing all quantities of GGBFS and NaOH powder in a dry form, where the reaction starts by “just adding water”. The other three mixes were designed using a microwave-chemical treatment method in which one-third of the used GGBFS (150 gm) and NaOH (45 gm) were put in a pottery plate, then sintered in a microwave oven at a power of 900 W for different durations (10, 20 and 30 min). Then the microwave-chemical treated powders (MCT) were quenched in the air to form an amorphous microstructure, followed by grinding to pass through a 75 μm sieve. The grinding process was conducted on MCT powder using a grinder with a power of 850 W for 3 min with a capacity of 300 gm per cycle. Finally, the ground MCT powder product was blended with two-thirds GGBFS (300 gm) to form OP-AAS powder. The preparation criteria for the microwave-chemically treated OP-AAS powder is presented in Fig. 2. The DM and OP-AAS fresh mixes were prepared by merging the powder with water, where the water/binder ratio (W/B) was determined in proportion to the standard water of consistency test as shown in Table ﻿3. Fresh pastes were transferred after mixing to one-inch cubic steel moulds and were cured at 23 ± 2 °C and 99 ± 1 percent relative humidity (RH) for 24 h, as recommended by several studies^82–86^. The hardened cubes were de-molded and cured in the same condition until the time of the test.

**Table 3:** Mixes preparation and design.

Mix ID	Blended treated powder			Residual mixed slag (gm)	Water/binder ratio
	GGBFS (gm)	NaOH (gm)	Microwaving (Min.) (900 W)		
TP	–	45	–	450	0.27
DM	150	45	–	300	0.25
OP-30M			30 min		0.35
OP-20M			20 min		0.365
OP-10M			10 min		0.365

### Fresh and hardened properties

The standard water of consistency, initial setting time (IST), and final setting time (FST) tests were determined using the Vicat apparatus according to ASTM (C191-19) and ASTM (C187-16). The mini-slump test is performed immediately after mixing to determine the flowability of the fresh pastes according to ASTM (C191-19). The test is performed by mixing the samples powder with water at a constant W/B ratio of 0.6, after which the fresh pastes were poured into a conical mould with dimensions: upper diameter = 19 mm, lower diameter = 38 mm, and height = 57 mm, then the cone was lifted upward vertically and the paste flowed with diameter directly proportional to mix flowability^87–89^. A high W/B ratio (0.6) was used to monitor the small change in the spread diameter. A compressive strength test was performed at curing ages 1, 3, 7, 28, and 56 days, according to ASTM (C109M-20b).

### Instrumental analysis

X-ray diffraction (XRD, Philips Xpert 2000) was used to monitor the effect of different treated microwaving periods on the phase composition of MCT powder and the mineralogical study of the binding phases formed during the hydration process. In addition, a scanning electron microscope (SEM, TESCAN VEGA 3) is used to characterize the morphology and microstructure of the zeolite-binding phases that help in the interpretation of the compressive strength results.

### Viability/cytotoxicity test

The effect of different concentrations of selected fresh mixes on human skin (HFP4 cells) with biological data shown in Table 4 was studied using the viability/cytotoxicity test according to the MTT protocol^90–92^. Initially, a 96-well tissue culture plate, with a volume of 100 μl/well, was inoculated with 104 cells/well and incubated at 37 °C for 24 h (Incubator, Memmert) to develop a complete monolayer sheet. After forming a gathered cell sheet, the growth medium was drained of the 96-well microtiter plates. The wash media is then used twice to wash the monolayer cell. RPMI medium with 2% serum (maintenance medium) is used to make two-fold dilutions of the sample tested. 0.1 ml of each dilution (31.25, 62.5, 125, 250, 500, 1000 ug/ml) for mixes (PC, TP, DM, OP-30M, OP-10M) was tested in 3 different wells, leaving three control wells without mixing samples, receiving only a maintenance medium. The plate was incubated at 37 °C for 24 h. Cells were examined for toxic physical signs of partial or complete loss of monolayer, deformation, rounding, or cell granulation.

**Table 4:** Biological data for HFP4 cells.

Organism	Home sapiens, human
Tissue	Foreskin
Cell type	Stellar, multipolar, or dendritic appearance; needle-like

MTT solution (5 mg/ml in PBS) (BIO BASIC CANADA INC) was prepared and then put into the cells, where 20 μl MTT solution was placed per cell to allow optical imaging of the viable residual cells using an inverted microscope (Nikon). The plate was placed on a shaking table for 5 min and 150 rpm to mix the MTT thoroughly into the media. To ensure MTT metabolization, the plate is incubated at 37 °C and 5% CO_2_ for 1–5 h. Then, the media is removed, and the plate is dried using paper towels to remove residue. The formazan (MTT metabolic product) is resuspended in 200 µl DMSO. To mix the formazan thoroughly with the solvent, the plate is placed on a shaking table for 5 min and 150 rpm. Optical density is read using the ELISA reader (Mindray MR-96A) at 560 nm and subtracting the background at 620 nm. The optical density should be directly correlated with the quantity of cells.

### Simplified economic and environmental impact

**Figure 3:**
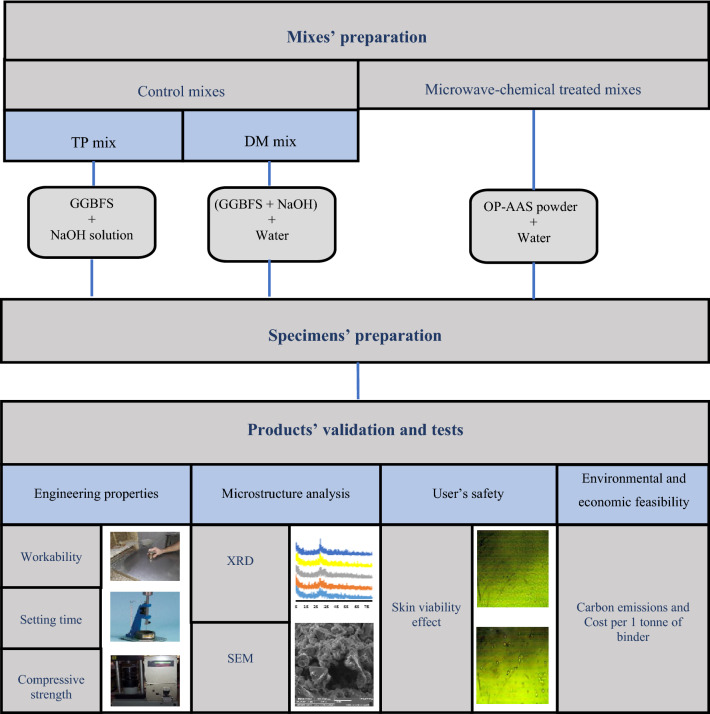
Experimental program stages.

To achieve more practical feasibility of the new product, two measures attributed to the CO_2_ emissions and the cost of sintered materials used to fabricate the geopolymeric binder were investigated. PC was added as a comparable commercial binding material. The environmental footprint of the mixes is obtained through the embodied CO_2_ emissions of the base materials used and the CO_2_ emissions produced during the microwave sintering and grinding process. The expected CO_2_ emissions for the used materials are 944, 26.5, and 1232 kg CO_2_/tonne for PC, GGBFS, and NaOH, respectively^93^. The CO_2_ emissions from a microwave oven and grinding process are 0.55 kg CO_2_/Kw h^94^. Also, the cost of base materials production ($/tonne) was calculated based on the Egyptian market prices. The cost calculation includes GGBFS and NaOH pellets prices in addition to the microwave sintering and grinding process costs. It was found that the average prices of the used base materials were 61.24, 41.15, and 208.32 $/t for PC, GGBFS, and NaOH pellets, respectively, while a 1 Kw h microwave oven and grinding process cost were 0.059 $/Kw h^60^. Table 5 demonstrates the amount of GGBFS, NaOH and microwave-chemical treated powders (MCT) as well as the amount of energy consumed during the microwave sintering and grinding process used to prepare 1 tonne of sintered materials employed in the fabrication of different geopolymeric mixes. Figure 3 presents a schematic diagram summarizing all the proposed experimental program stages.

**Table 5:** Amount and type of ingredients as well as energy consumed for preparation of 1 tonne of sintered materials used to fabricate geopolymeric binders.

Technique	Ingredients	Base materials (Weight, Kg)				Treatment period	E_MV_ (Kw h)/tonne	E_G_ (Kw h)/tonne
		Without treatment			With treatment			
		pc	GGBFS	NaOH	33.3% GGBFS + NaOH			
Commercial	PC	1000	–	–	–	No treatment applied	No additional energy applied	
Two-part	GGBFS + NaOH	–	909	91	–			
Dry-Mix								
One-part uses a microwave-chemical treatment process	GGBFS + NaOH	–	606	–	394	10 min	394	55.8
						20 min	788	55.8
						30 min	1182	55.8

E_MV_ and E_G_: is the energy consumed during the microwave sintering and grinding process used to prepare 1 tonne of sintered material used in the preparation of one-part geopolymer.

## Results and discussion

### Thermo-chemical treated GGBFS characterization

Different microwave-chemical treated powders (MCT) were formulated by microwave-chemical activation of GGBFS blended with NaOH powder for different treatment periods, followed by quenching to generate cementitious vitrified material with high calcium and sodium content as well as an amorphous aluminosilicate source. Different microwaving treatment periods (10, 20 and 30 min) at 900 W and 10 wt.% NaOH was examined to determine the optimum condition for producing highly reactive aluminosilicate materials. XRD analysis was applied to evaluate the effect of the treatment period on the amorphous content of the produced materials.

**Figure 4:**
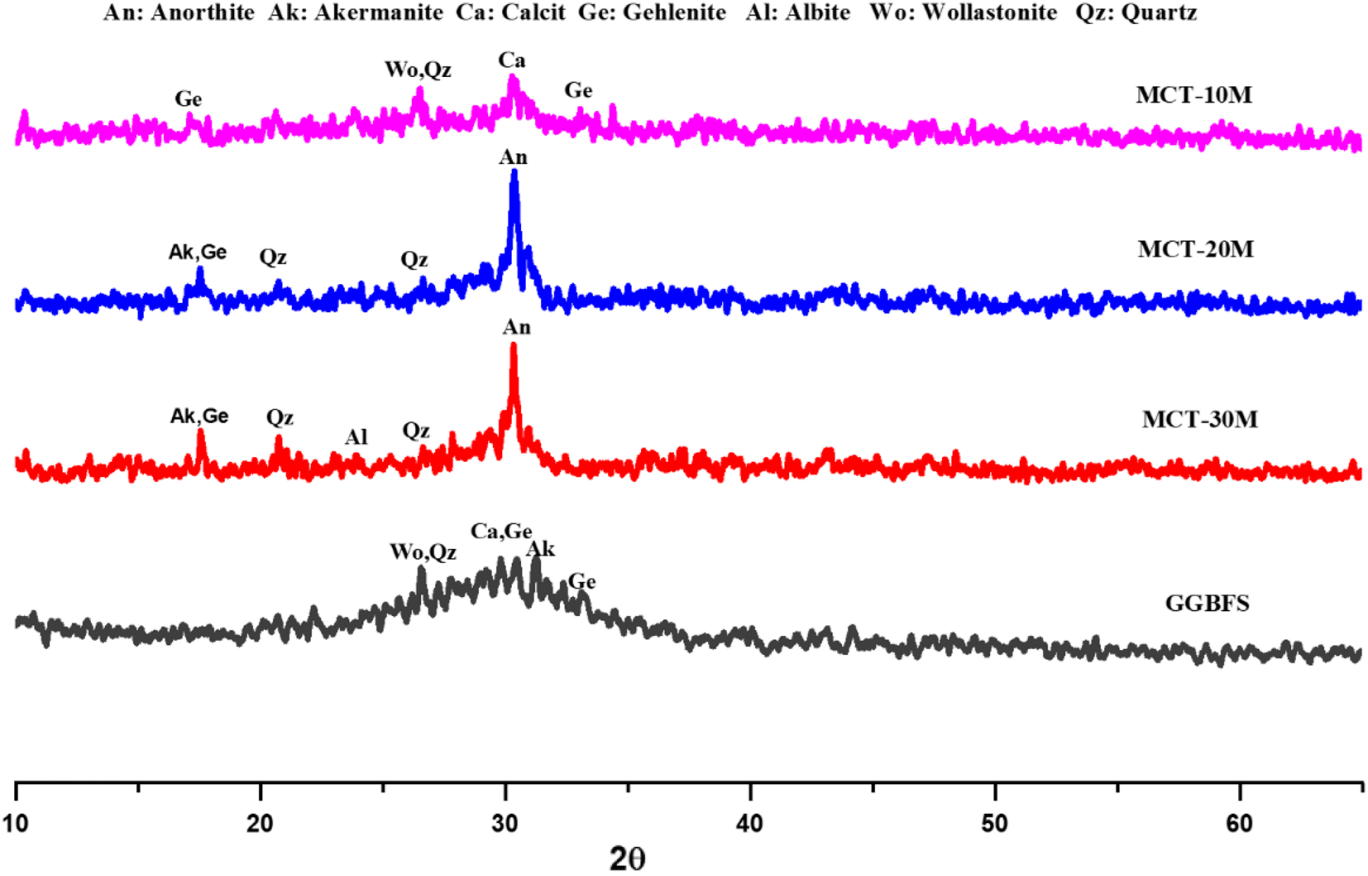
XRD-patterns of GGBFS and thermochemically treated powders.

XRD pattern in Fig. 4 presented an amorphous microstructure of GGBFS with a wide hump at 22.6°–37.9° 2θ containing semi-crystalline peaks with a low intensity related to wollastonite (CaSiO_3_, PDF# 00-043-1460) and Quartz (SiO_2_, PDF# 01-087-2096) at 26.6° 2θ, calcite (CaCO_3_, PDF# 01–088-1808) at 29.8° 2θ, gehlenite (Ca_2_Al_2_SiO_7_, PDF# 01-079-2423) at 29.81 and 33.1° 2θ, and akermanite (Ca_2_MgSi_2_O_7_, PDF# 01-079-2424) at 31.3° 2θ^95–97^. After microwave treatment of GGBFS for 10 min in the presence of NaOH (MCT-10M), the XRD pattern showed a highly amorphous microstructure with decreasing in the intensities of akermanite and gehlenite crystalline phases. The dissolution of akermanite and gehlenite phases clarifies the fluxing ability of NaOH in the depolymerization of crystalline aluminosilicate materials network and forms an amorphous structure with high sodium ion existence^57,98,99^. Increasing the microwave treatment period to 20 min (MCT-20M) leads to the microstructure recrystallization and forming of new peaks of gehlenite (Ca_2_Al_2_SiO_7_, PDF# 01-079-2423) overlapped with akermanite (Ca_2_MgSi_2_O_7_, PDF# 01-079-2424) at 17.55° and anorthite (Ca(Al_2_Si_2_O_8_), PDF# 01-073-0265) at 30.29° 2θ. By increasing the microwave treatment period to 30 min (MCT-30M), crystalline peaks were observed, which refer to quartz (α-SiO2, PDF 01-079-1910) at 20.63°and 26.53° and refer to albite (Na[AlSi_3_O_8_], PDF 00-041-1480) at 23.81°, and 34.39° 2θ^57,100,101^. XRD pattern results illustrated the effect of increasing the treatment period on forming stronger bonds and phases between NaOH and GGBFS. Also, the results showed the crystalline phase formation that affects the activity and hydration ability of the mix, which clarifies the negative effect of a long microwaving treatment period on active amorphous materials such as GGBFS.

### Mini slump test

**Figure 5:**
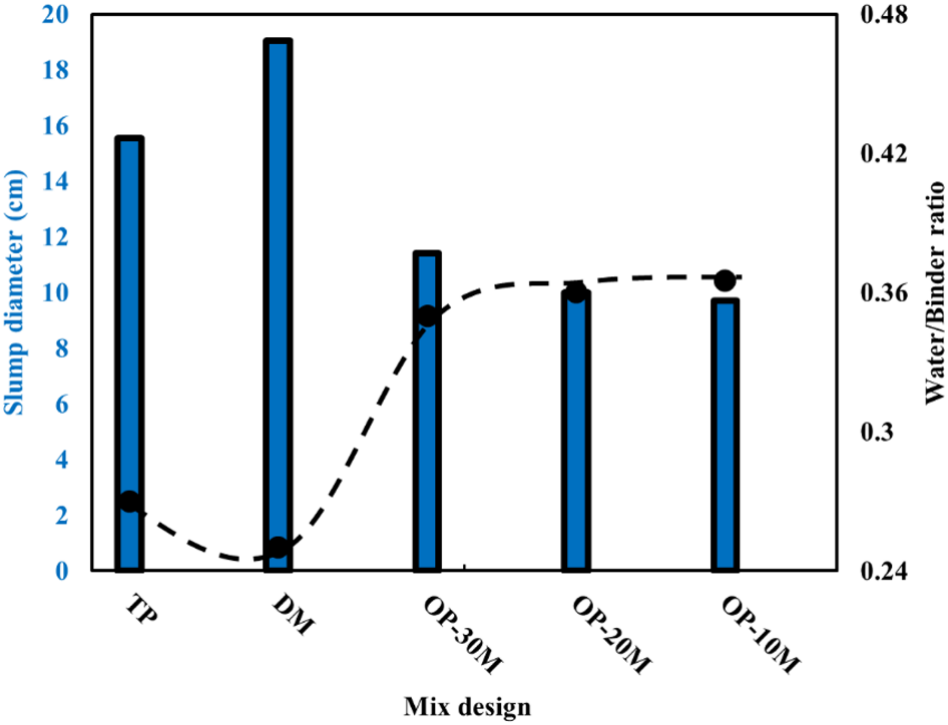
Mini slump/Water consistency values for TP, DM and OP-AAS.

Workability is the property of fresh pastes that defines the easiness by which it can be mixed, placed, and finished as defined by ACI Standard 116R-00 (ACI 2000). The mini-slump test values for TP, DM, OP-30M, OP-20M, and OP-10M are given in Fig. 5. It is observed that DM and OP-AAS mixes showed different slump results that clarify the role of the microwave-chemical treatment process in varying the workability behaviour for the treated mixes. The initial high slump value for DM is due to the time taken to dissolve NaOH powder in water before the beginning of the chemical reaction, followed by the exothermic reaction. While in the case of the TP mix, NaOH was already dissolved in water before mixing, so once the water was added, the reaction was initiated. For OP-AAS mixes, it is observed that the mini-slump values increased by increasing the microwave treatment period. This behaviour can be explained as follow: increasing the microwaving treatment period was accompanied by an increase in the degree of crystallinity as clarified by XRD. This crystallinity decreased the hydration ability of the mix and hence more water was enabled to achieve higher slump values^102,103^. Furthermore, as the crystallization increased, a bond was formed between sodium oxide and the base material, which decreased the amount of free sodium oxide, delaying the hydration process and giving a chance for better workability. Therefore, it is concluded that there is a direct relationship between the mixes' reactivity and the rate of loss in workability.

### Water of consistency test

The water of consistency test gives the proper W/B ratio for the paste to give optimum homogeneity in terms of strength and workability^104^. Figure 5 shows the water consistency values for TP, DM, OP-10M, OP-20M, and OP-30M. Although all share the same ratio of the base material, the water requirement for each mix differs according to the way of mixing. For microwave-chemical treated mixes OP-10M, OP-20M, and OP-30M, it was observed that the required water for hydration decreased by increasing the microwave treatment period from 10 to 30 min. This decrease indicates the formation of more inactive and crystalline phases, which decreases the mix hydration ability. DM shows a low need for mixing water, although high heat is released after mixing. This behaviour is due to the dissolution of NaOH in water occurring first before the geopolymerization reaction begins, which makes W/B = 0.25 sufficient for achieving the penetration value without sharing any water in the reaction. All standard water of consistency test values is aligned with mini-slump and setting time test values as will be illustrated.

### Setting time

**Figure 6:**
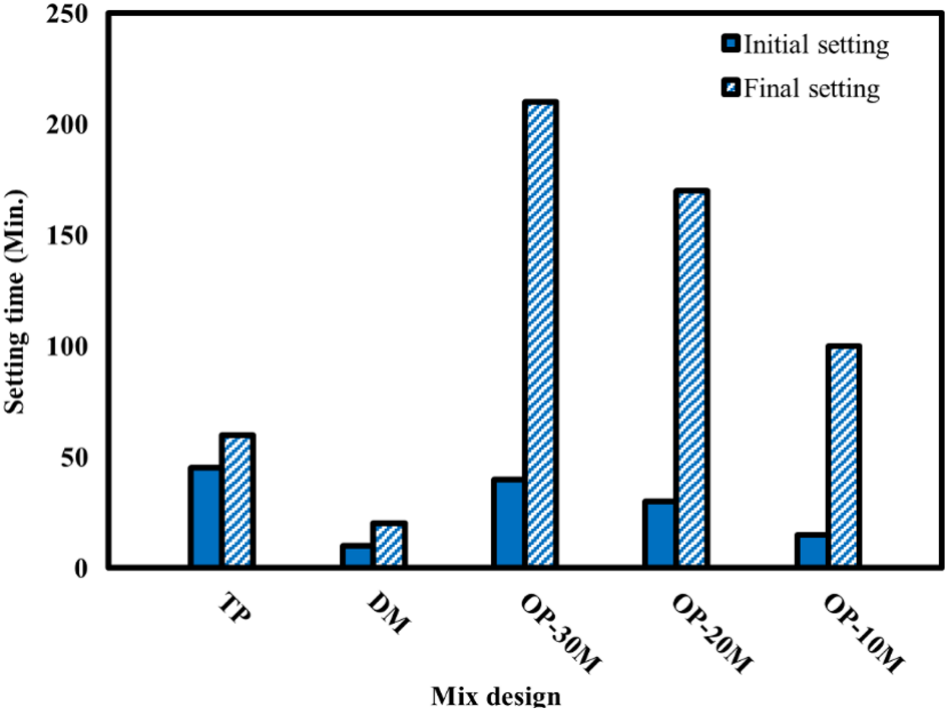
Setting time values for TP, DM and OP-AAS mixes.

Setting time is an essential property for the binding materials, which indicates the beginning of the hydration process. Setting time should be within a certain limit, which is not too short to allow mixing, casting, and finishing processes and not too long to allow formwork and mold releasing. IST and FST values for TP, DM, OP-10M, OP-20M, and OP-30M are given in Fig. 6. TP and DM results show different setting behaviour, which can be explained by the dissolution mechanism of NaOH powder in water. In the case of DM, the heat resulting from the reaction between NaOH powder and water increased at the rate of the geopolymerization process. Also, it was observed that the setting time results for the microwave-chemical treated mixes are affected by the microwave treatment period. As the treatment period increases from 10 to 30 min, the setting time increases significantly. This increase is due to the bond formed between Na ions and the aluminosilicate materials, which strengthened with the treatment period and retards the chemical reaction to begin^103^.

### Compressive strength

**Figure 7:**
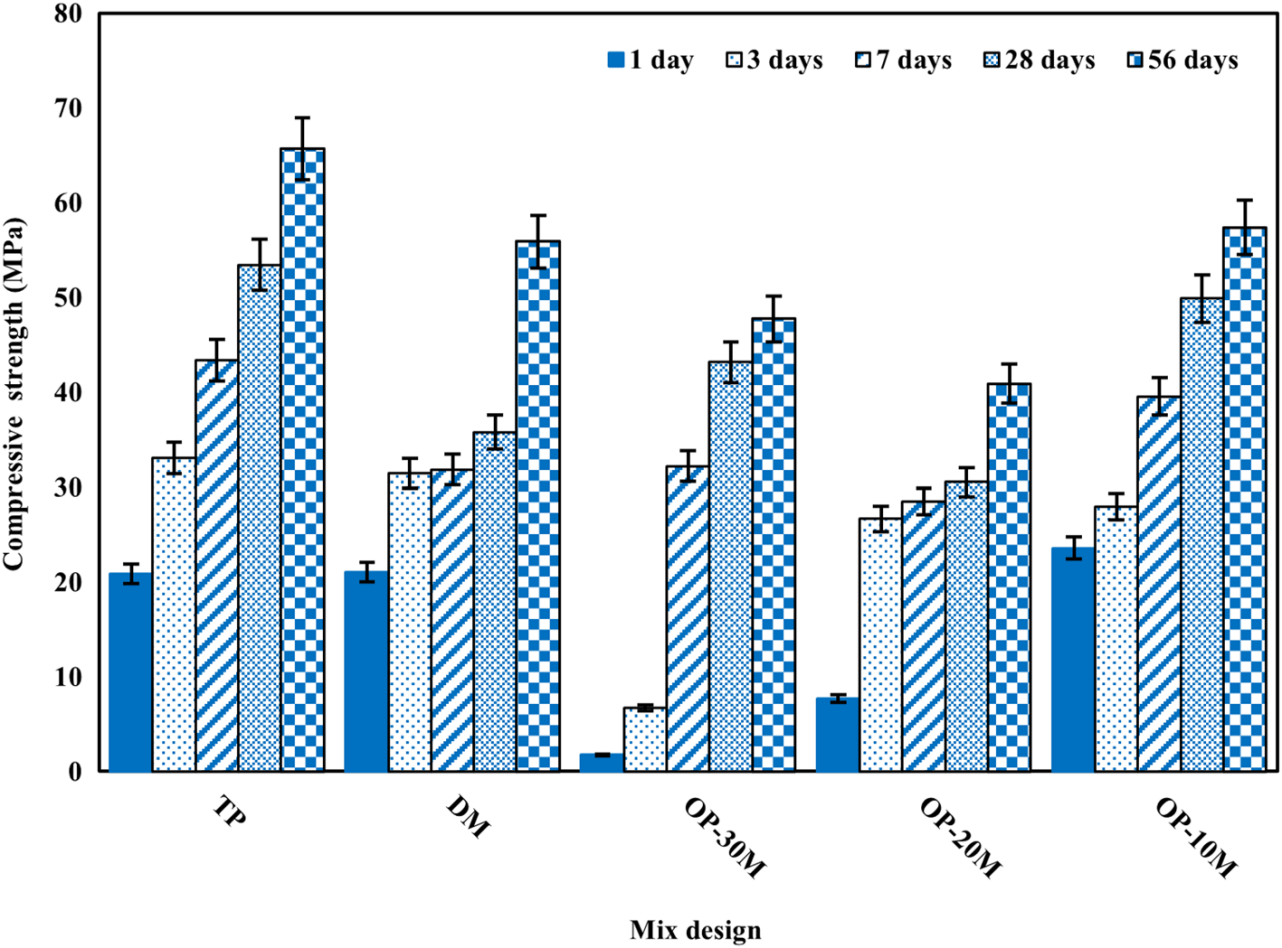
The effect of sintering temperature on Compressive strength.

The principal role of microwaving of the material is the thermal excitement of bonds between its internal particles. The presence of NaOH (fluxing material) with the microwaving process worked together to increase the amorphousity of the aluminosilicate material. After adding water to the OP-AAS powder, hydration begins by liberating bonded Na cations from the treated powder to build the free NaOH alkali. Then, following the same steps as the two-part AAB of dissolution, condensation, and polymerization, forming hardened material with adequate compressive strength^57,58,105,106^. The mechanical compressive strength (MCS) development of TP, DM, OP-30M, OP-20M, and OP-10M is given in Fig. 7. MCS increases with increasing the curing time because of the development of hydration and geopolymerization process and the continued formation of hydration products such as calcium silicate hydrate (C–S–H), calcium–aluminium–hydrate (C–A–H), and calcium–aluminosilicate–hydrate (C–A–S–H).

On 1 day of curing, TP and DM show high early strength, which can be explained by the presence of Na ions in their free state, which promotes the dissolution of aluminosilicate networks and the geopolymerization process^86,95,107^. For OP-AAS mixes, it was observed that the MCS behavior changed for each mix by changing the microwaving period. OP-30M and OP-20M show low early MCS compared with TP and DM mixes, which clarify the role of microwaving in mitigating the effect of the alkaline activator by bonding Na ions to the aluminosilicate structures by strong bonds, which retard Na ions' liberation rate, hence the geopolymerization process^62^. This retardation depends on the strength degree of bonds formed between Na ions and the aluminosilicates, which depend mainly on the microwaving treatment period. OP-10M shows a significant early strength compared with TP and DM mixes. This early strength indicates the high reactivity of the mix and clarifies the sufficiency of 10 min microwaving treatment period to only bond the Na ions to the aluminosilicates with weak bonds. These weak bonds did not affect the progress of the geopolymerization process.

On 3 days of curing, the geopolymerization reaction continues with a remarkable increase in compressive strength values for OP-20M and OP-10M. Low MCS development was observed for OP-30M. On 7 days of curing, hydration proceeds for all mixes with a significant regression in MCS development for the DM mix. This regression is due to the micro-cracks formed due to high heat released during the exothermic reaction of dissolution of NaOH in water. OP-30M shows a notable increase in MCS value. This late start indicates that the degree of crystallinity increases as the microwave treatment period increases. The Na2O is strongly bonded to the alkali-activated powder, which needs more time to become free to form the highly alkaline medium needed for the geopolymerization process.

On 28 days of curing, geopolymerization process development is proceeding for all mixes. DM still shows hydration development regression due to suffering from microcracks formation. On 56 days of curing time, OP-10M shows a highly significant MCS compared with TP and DM, which refers to the possibility of preparing OP-AAS with high MCS, mitigating the alkaline activator's harmful effect, and using low energy.

### X-ray diffraction test

**Figure 8:**
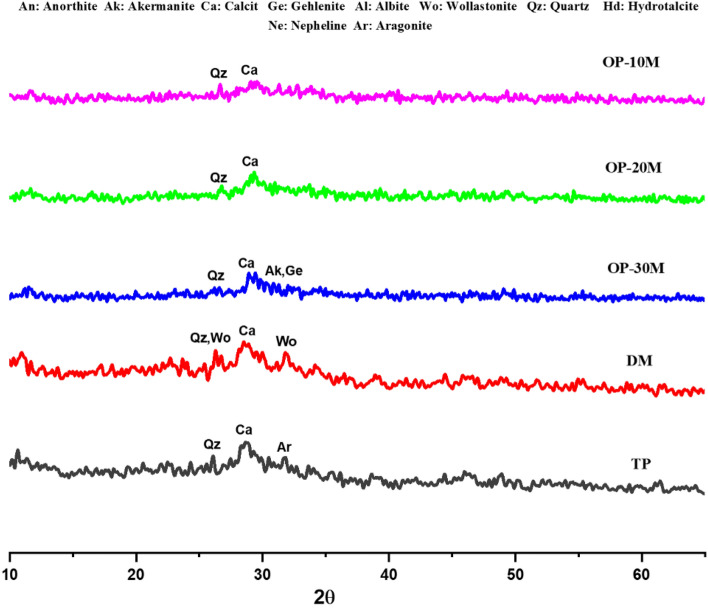
XRD-patterns for TP, DM and OP-AAS mixes after 1 day of hydration.

**Figure 9:**
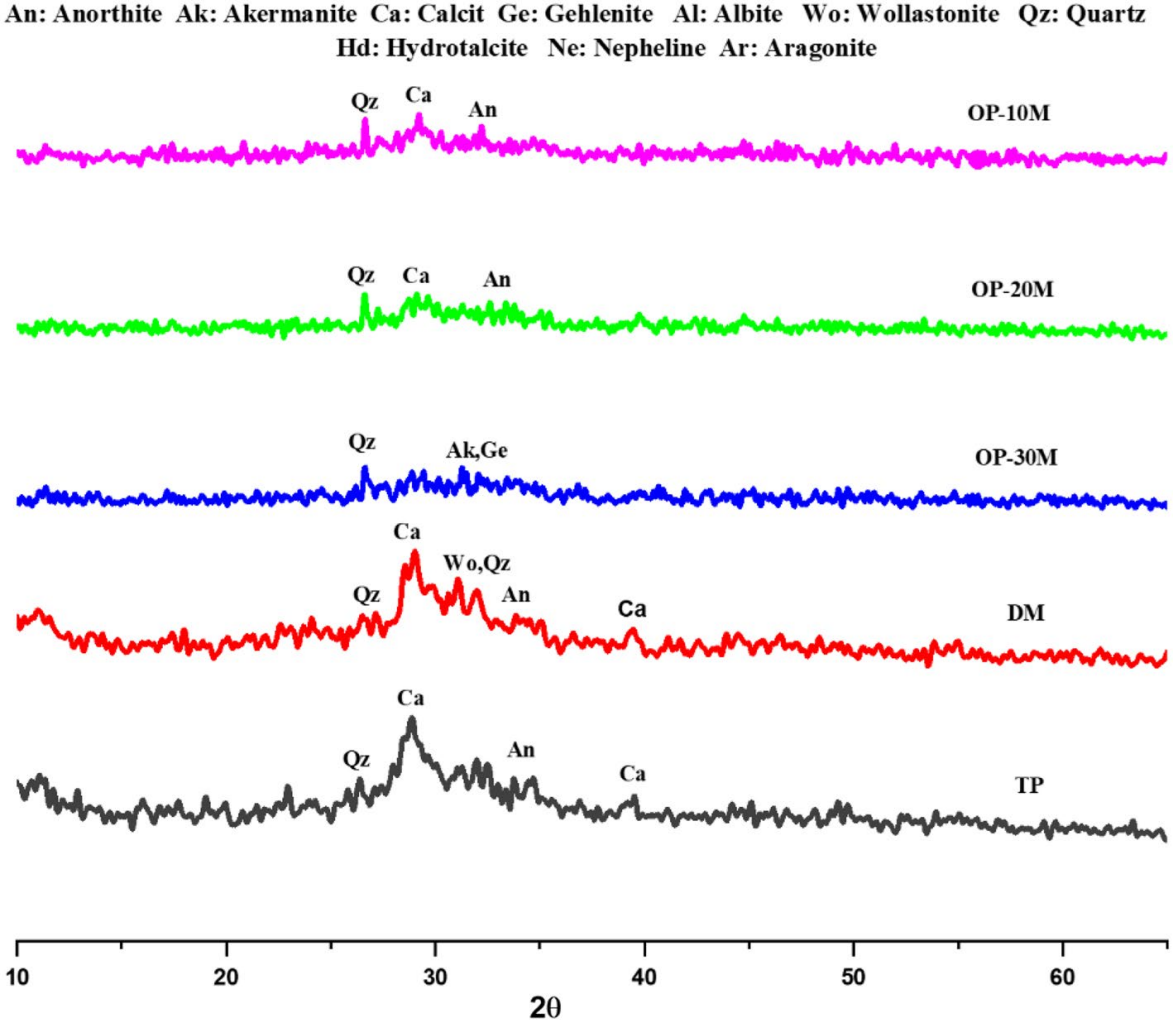
XRD-patterns for TP, DM and OP-AAS mixes after 28 days of hydration.

The XRD patterns of hydrated TP, DM, OP-30M, OP-20M, and OP-10M mixes at curing ages (1 and 28 days) are shown in Figs. 8 and 9. It was revealed that all the hydrated mixes show the same peaks with different intensities in addition to the appearance of new peaks depending on the mix's internal characteristics and curing duration. The hydration products of all mixes noticed were ill-crystalline and amorphous phases with the presence of a small number of crystalline peaks, which points to the creation of binding phases. On 1 day of curing, Fig. 8, TP mix showed a broad hump ranging between 24.78–33.61° 2θ that centered at 28.93° 2θ related to calcite (CaCO_3_, PDF 01-071-3699)^95^, ill-crystalline from tobermorite-phase (C–S–H, PDF# 00-033-0306) in addition to Al-tobermorite-phase (C–A–S–H, PDF# 00-020-0452) as hydration products^42,95,108,109^. The same peaks were observed in the DM mix with the presence of intense high peaks at 26.3° 2θ referring to quartz (SiO_2_, PDF# 01-087-2096)^110^ in addition to the wollastonite phase (CaSiO_3_, PDF# 00-043-1460) at 26.3 and 31.9° 2θ^95,111–113^, which highlights the need for NaOH powder dissolution to be suitable for activation of the phases present in GGBFS. In the OP-30M mix case, a reduction in the intensity of the broad hump was observed, which indicates the low reactivity of alkali-activated powder treated for 30 min. Also, peaks were observed, which represent quartz (α-SiO2, PDF 01-079-1910) at 26.65°, akermanite (Ca_2_Mg[Si_2_O_7_], PDF 01-079-2424) and gehlenite (Ca_2_Al[AlSiO_2_], PDF 01-079-2423) at 31.31° 2θ^95,114^. These peaks refer to the presence of a high percentage of unreacted GGBFS phases. The results are in line with MCS and SEM results. Nevertheless, the broad hump in the OP-20M mix is more clearly shown than in the OP-30M mix. In OP-10M, a noted increment in the broad hump was observed with the presence of calcite (CaCO_3_, PDF 01-071-3699) peak at 29.21°, which refers to the formation of a high amount of binding phase. The new binding phase formed explains the high amorphousity and activity of the alkali-activated powder and the significant compressive strength values for the OP-10M mix.

On 28 days of curing, most of the abovementioned zeolitic binding phases are identified in Fig. 9. The intensity of these peaks in all mixes was remarkably increased, which refers to continuing the geopolymerization process with time, confirming the results of MCS values.

### Microstructure

**Figure 10:**
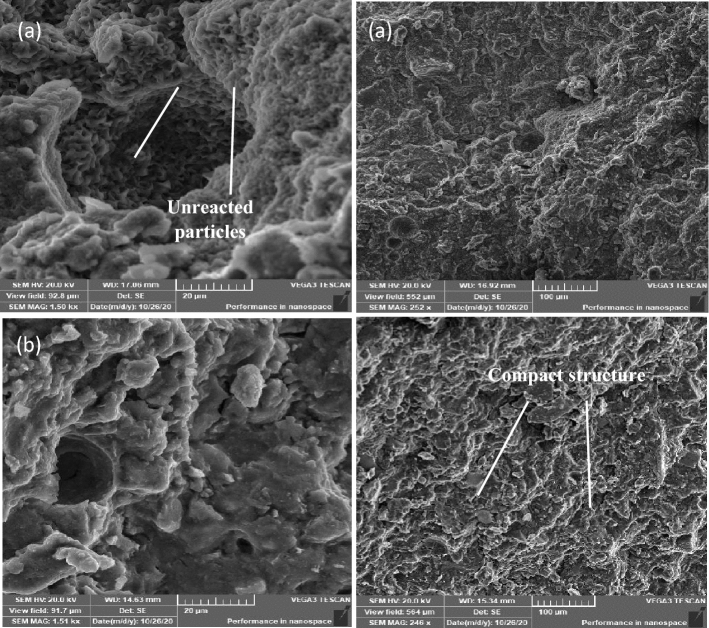
SEM for TP mix. (**a**) 1-day (**b**) 28 days.

**Figure 11:**
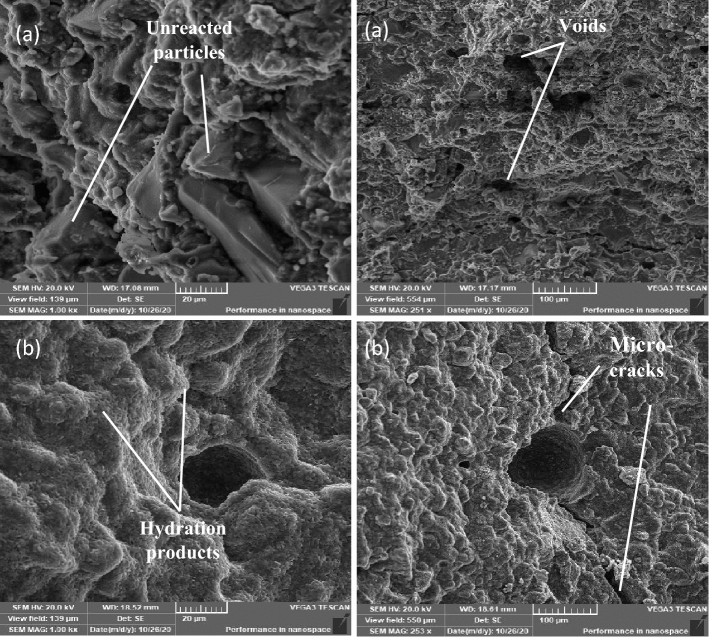
SEM for DM mix. (**a**) 1-day (**b**) 28 days.

**Figure 12:**
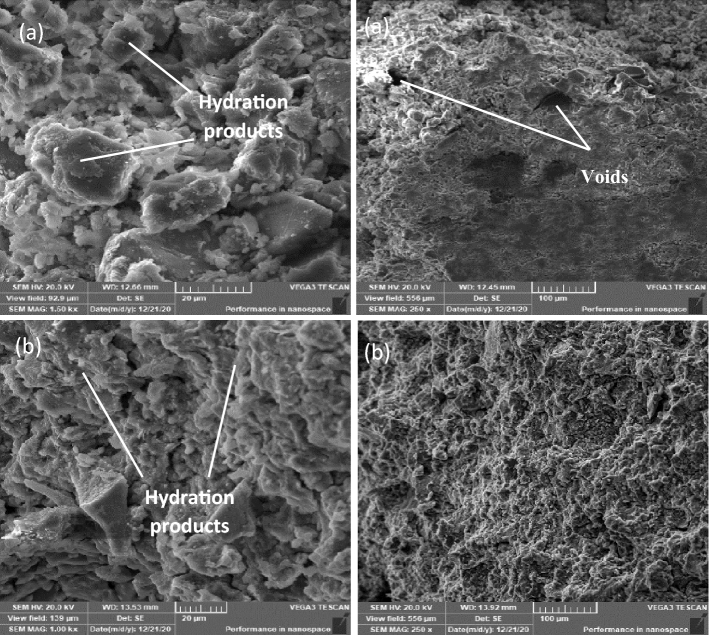
SEM for OP-10M mix. (**a**) 1-day (**b**) 28 days.

**Figure 13:**
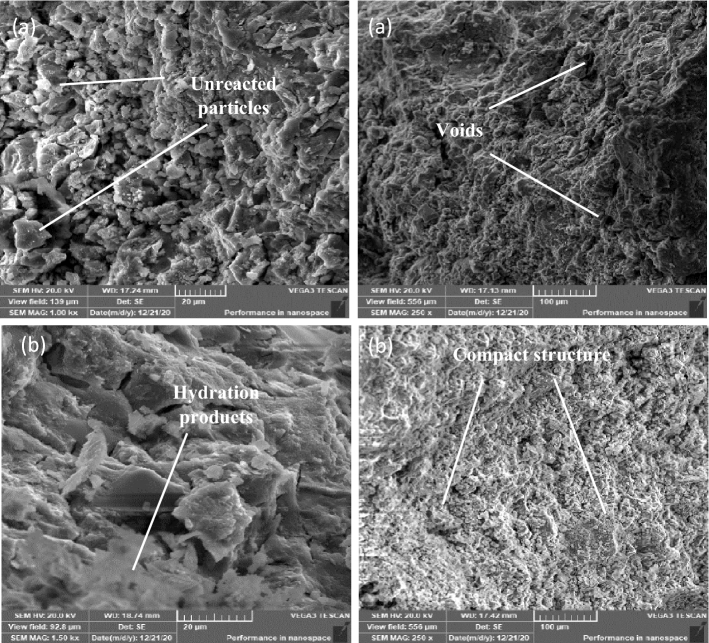
SEM for OP-30M mix. (**a**) 1-day (**b**) 28 days.

SEM examination for mixes (TP, DM, OP-30M, OP-10M) on 1 day and 28 days of curing time is represented in Figs. 10, 11, 12 and 13, respectively. The SEM- micrograph for the TP and DM mixes clarifies the effect of the activator nature (NaOH solution or NaOH solid powder) on the behaviour of the geopolymerization process of GGBFS. Figures 10a and 11a show a 1 day micrograph of TP and DM mixes. The images show a good compact, dense microstructure due to the formation of the tobermorite gel of (C-S–H) and products of (C–A–H, and C–A–S–H)^115,116^. On 28 days of curing, Figs. 10b and 11b show a more compact microstructure with notable micro-cracks of DM mix, which explains the low MCS that is 45% lower than the TP mix. These micro-cracks could result from internal stresses induced by the high amount of heat that evolved, in the early age, during the dissolution of NaOH powder in water, as reported by Xiang et al.^54^, and Shen et al.^55^. Also, Lima et al.^117^, and Collins et al.^118^ reported that the micro-cracks are due to autogenous shrinkage and the creation of voids by the dissolution of metasilicate, which consequently exert internal stresses and micro-cracks occurred.

Figs show SEM images for OP-10M and OP-30M for microwave-chemical treated mixes. (12) and (13), respectively. On 1 day of curing, OP-10M showed a good compact microstructure, with the formation of hydration products (Fig. 12a). OP-30M showed a Highly disordered microstructure with high porosity compared to TP and DM mixes with the presence of a high number of unreacted particles with no existence of hydration products Fig. 13a. On 28 days of curing, both OP-10M and OP-30M show a more organized and denser microstructure with observing hydration products of g as shown in Figs. 12b and 13b. The variation in behaviour between OP-10M and OP-30M highlights the effect of the microwave treatment period on the internal microstructure of the mixes.

### Viability/cytotoxicity test

**Figure 14:**
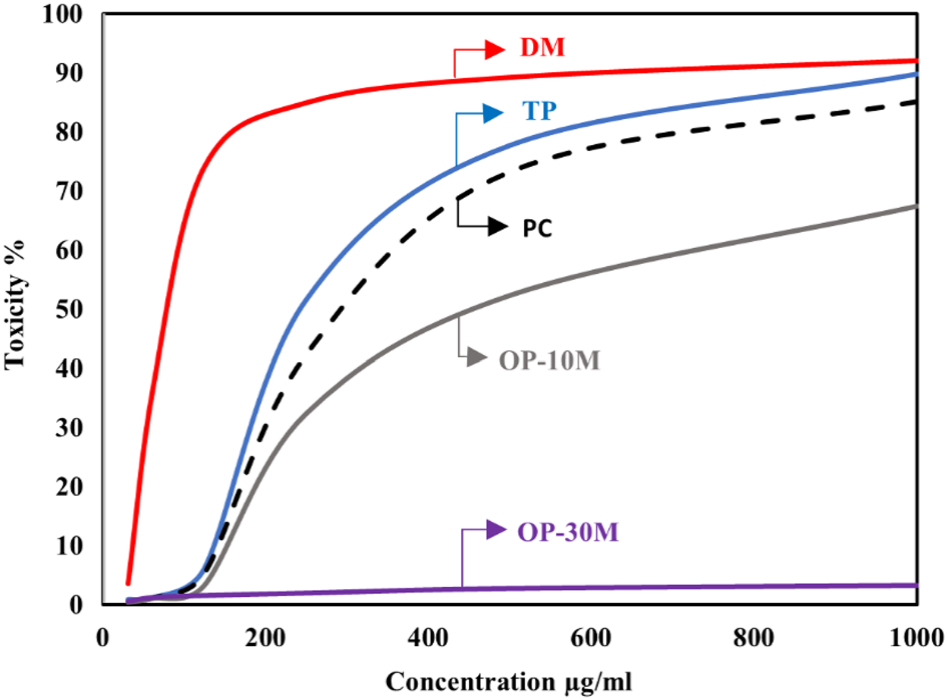
The toxicity effect of TP, DM, OP-AAS and OPC with different concentration on HFP4 cells.

Toxicity is the ratio between unviable cells in the well exposed to the tested mixes and the viable cells that exist in the control well. Cytotoxicity test results for PC, TP, DM, OP-30M and OP-10M mixes at different concentrations are shown in Fig. 14. Generally, the concentration of added mixes to the viable cell, the toxicity increased. TP and DM samples showed the highest toxicity in the AAM, which caused damage of 50% from the viable cells with a concentration of 245 µg/ml and 89 µg/ml, respectively. The high toxicity of TP and DM mixes was because of the presence of the alkaline activator in a free state, which is harmful, toxic, and a skin irritator. The difference in the toxicity degree between DM and TP, shown in Fig. 14, was due to the high heat released, in the case of DM, from the exothermic reaction of dissolution of NaOH in water, which caused more damage to the viable cells. PC showed medium toxicity compared to TP and DM mixes, as 305 µg/ml of PC was enough to damage 50% of the cells. For thermo-chemically treated mixes (OP-30M and OP-10M), the results showed the treatment period's high effect on the tested mixes' toxic behavior.

**Figure 15:**
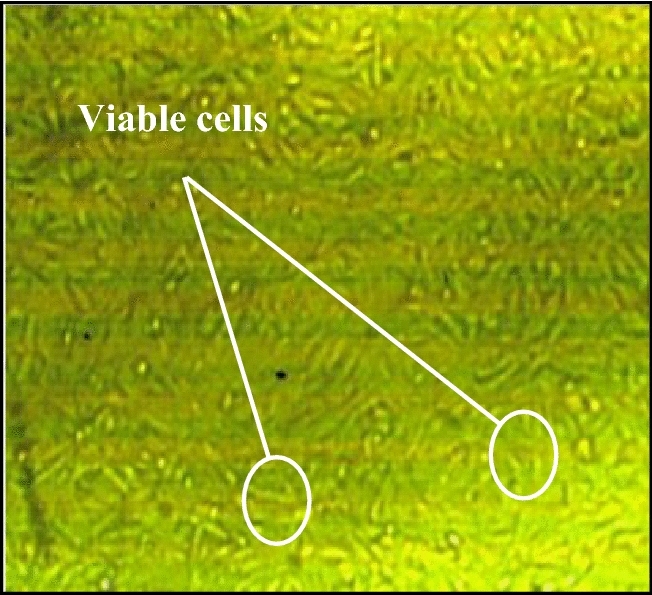
Control well image with HFP4 cells.

**Figure 16:**
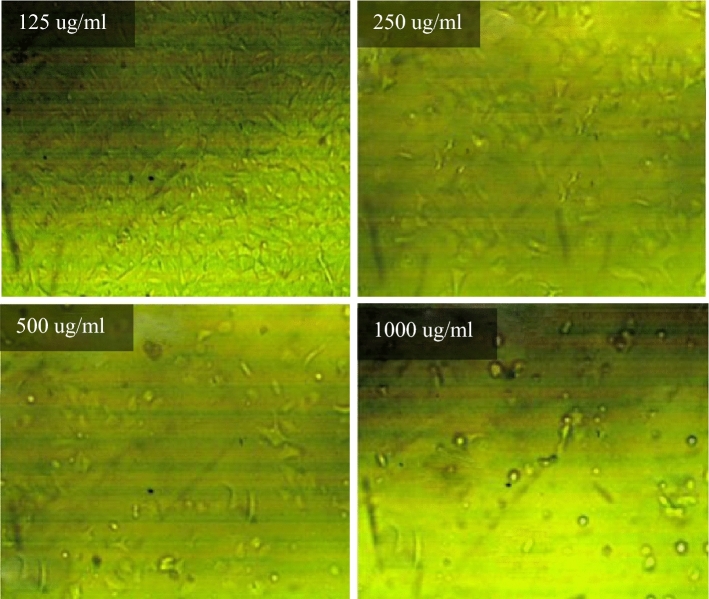
Optical images for TP samples with different dilutions.

**Figure 17:**
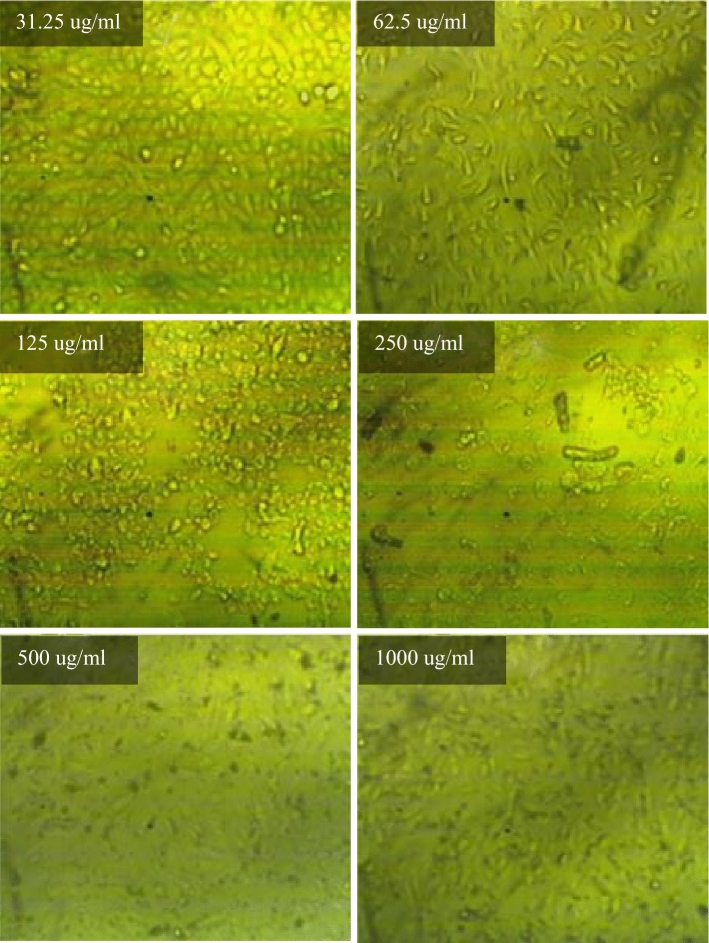
Optical images for DM samples with different dilutions.

**Figure 18:**
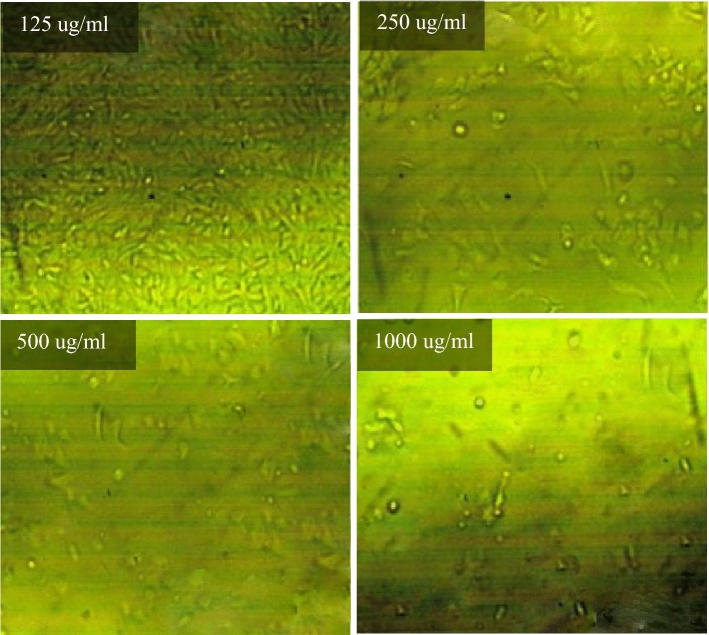
Optical images for OPC samples with different dilutions.

**Figure 19:**
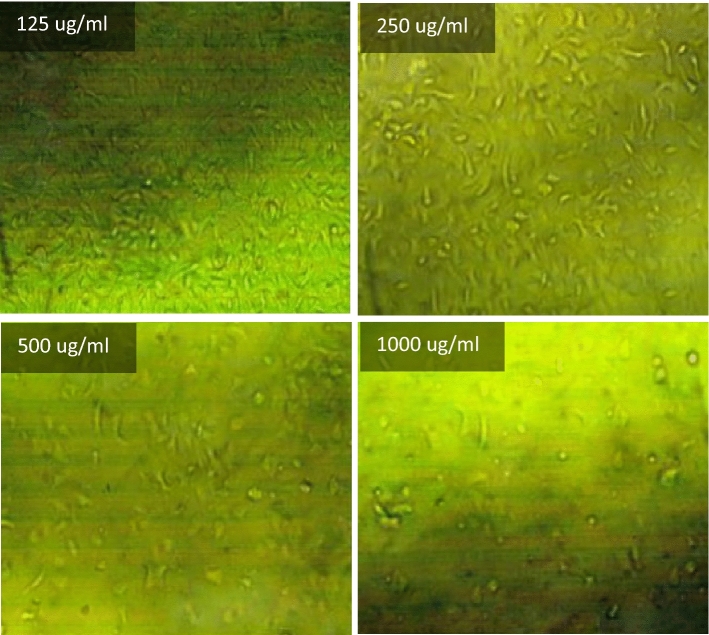
Optical images for OP-10M samples with different dilutions.

**Figure 20:**
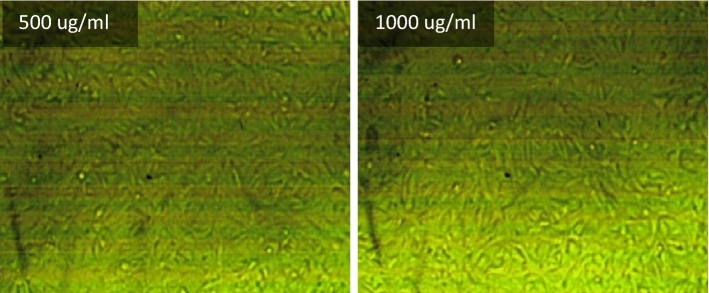
Optical images for OP-30M samples with different dilutions.

By increasing the treatment period, the toxicity of the samples decreased, as shown in Fig. 14. This remarkable decrease in toxicity clarified the ability of the thermal energy to combine the alkaline activator with the base material (GGBFS) and mitigate the harmful effect of the alkaline activator. The binding percentage developed gradually with an increment of the treatment period until a transition period (related to NaOH’s melting point = 318 °C). After this transition period, NaOH became more embedded into the aluminosilicate precursor, and the toxic effect of pastes decreased and totally vanished, as shown for OP-30M. The optical images of remaining cells before and after exposure to TP, DM, OP-30M, OP-10M, and PC samples were shown in Figs. 15, 16, 17, 18, 19 and 20. It is clarified that there is a direct relationship between the decrease in the number of viable cells and the toxicity of the mixes. Comparing the optical images of these mixes with control cells, the disappearance of viable cells was observed in DM and TP (Figs. 16 and 17) and partial disappearance in PC and OP-10M (Figs. 18 and 19). However, there was no remarkable variation in the number of viable cells in the case of OP-30M Fig. 20. Finally, it is concluded that an optimum OP-AAM mix with high energy efficiency and safe to use by laborers can be prepared through a microwaving period ranged from (10 min to 30 min).

### Embodied CO_2_ impact and cost calculation

**Figure 21:**
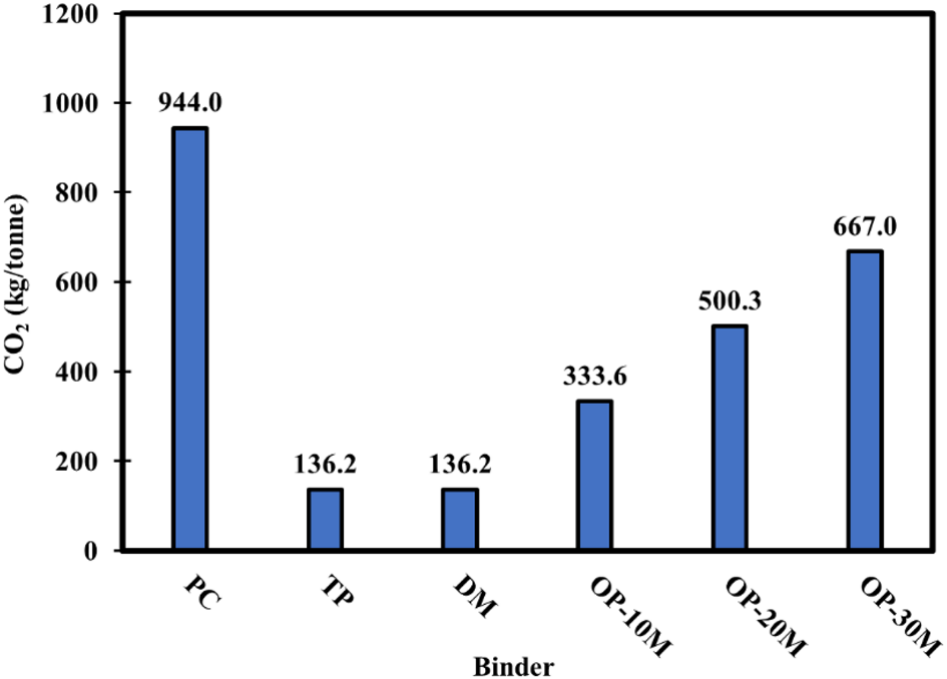
Calculated carbon emissions of 1 tonne PC, TP, DM, OP-AAS binders.

A product’s environmental footprint is one of the main measures to be considered when choosing alkali-activated materials as a sustainable alternative to commercial binder (PC)^119–121^. Figure 21 represents the simplified CO_2_ emission values per tonne produced from manufacturing materials fabricating PC, TP, DM, and OP-AAS binders. Generally, the PC binder showed much higher CO_2_ emissions than all the alkali-activated binders (TP, DM, OP-AAS). This tangible difference reflects the role of utilizing alkali-activated binders as sustainable alternatives to PC in the construction sector. On the other hand, the control binders (TP, DM) show a low carbon footprint of about 14.42% than PC. Regarding OP-AAS binders, it was observed that the microwave-chemical treatment process significantly affects the CO_2_ emission values. By using microwave treatment, the total CO_2_ emissions of the sintered materials manufacturing used in OP10M, OP20M and OP30M binder reached 35.33, 53 and 70.65% of that of the PC binder, respectively.

**Figure 22:**
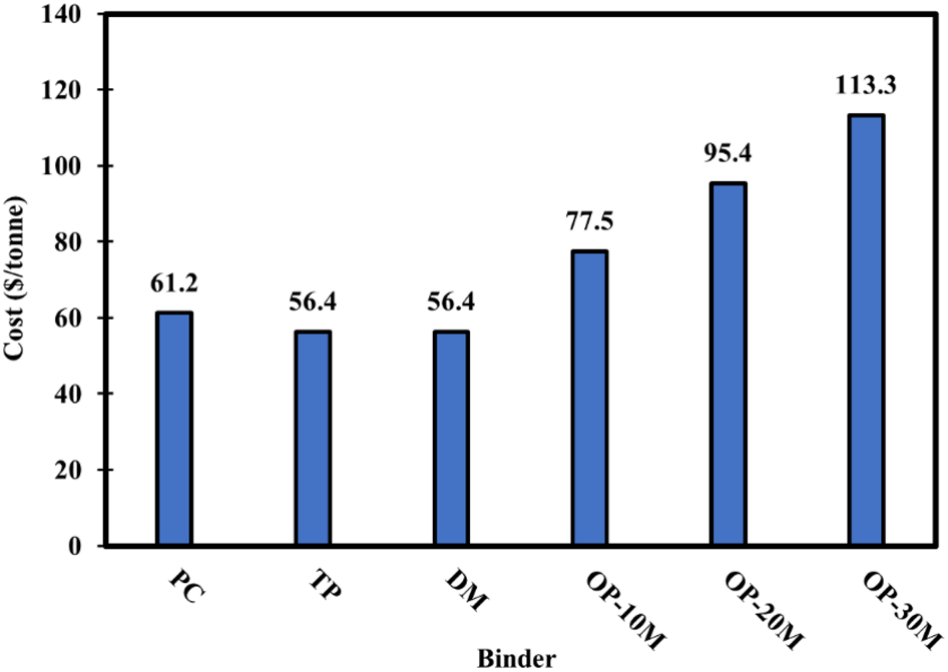
Calculated costs of 1 tonne PC, TP, DM, OP-AAS binders.

The cost of products is highly dependent on different factors, such as the availability of raw materials and product scalability. Accordingly, the mass production of such OP-AAS binders will be expected to contribute to an additive lowering in the binder's price. Figure 22 represents the simplified costs per tonne required for producing materials used for manufacturing PC, TP, DM, and OP-AAS binders. Generally, proposed alkali-activated binders (GGBFS + NaOH) have an approximately equal cost of PC due to the relatively expensive cost of NaOH compared to PC and GGBFS. The microwave sintering process and grinding of sintered materials contributed to a significant increase in the cost of OP-AAS binders compared to control binders (TP, DM). By increasing the treatment period from 10 to 30 min, the cost of manufacturing 1 tonne of OP10M, OP20M and OP30M binders increased by 26.63, 55.88 and 85.13% than PC binder cost, respectively.

## Conclusion

The main motivation for this study is employing microwave sintering to minimize the energy required to prepare one-part AAB. The microwave was used as a clean energy source to develop sustainable and eco-friendly binders. Based on the experimental study results and analysis, the conclusions can be pointed out as follows:

1. It is possible to produce a sustainable one-part AAB with adequate compressive strength using active amorphous GGBFS and a low-energy microwave-chemical treatment process.
2. Increasing the microwave treatment period led to a decrease in the amorphous nature and then the reactivity of the MCT powder, which is highly affected on the fresh and hardened properties of the OP-AAS. When the curing period increased from 10 to 30 min, the initial and final setting was increased by 166.67 and 110%, respectively, the workability was increased by 17.53% and the compressive strength was decreased by 11.95% at 28-days.
3. Retardation in early compressive strength values of OP-AAS, especially that prepared from MCT-30M compared to TP and DM specimens, refers to the impact of microwave sintering in binding the alkaline activator to the base material (GGBFS) as confirmed by the formation of new phases in the XRD analysis of MCT powder. Consequently, the alkaline activator becomes not free to start the geopolymerization process.
4. Applying microwave chemical treatment on the base material mitigates the effect and threats of the alkaline activator on users' and laborers’ skin, as reported by the cytotoxicity test results. All OP-AAS mixes show a lower effect on the skin; also, their effect decreases by increasing the treatment period from 10 to 30 min due to the embedding of NaOH in the base materials.
5. Although employing the microwave for producing sintered materials used in the fabrication of OP-AAS comparatively has a high cost than PC, reliance on OP-AAS as alternative binding materials to PC is recommended due to its low CO_2_ emission. Also, despite the CO_2_ emission and cost of TP and DM being lower than OP-AAS, the production of OP-AAS will solve the handling problem of alkali-activated materials resulting from the alkaline activator's harmful, toxic, and skin-irritating effect

## Data Availability

All data generated or analysed during this study are included in this published article.
